# Alterations in the gut bacterial microbiome in people with type 2 diabetes mellitus and diabetic retinopathy

**DOI:** 10.1038/s41598-021-82538-0

**Published:** 2021-02-02

**Authors:** Taraprasad Das, Rajagopalaboopathi Jayasudha, SamaKalyana Chakravarthy, Gumpili Sai Prashanthi, Archana Bhargava, Mudit Tyagi, Padmaja Kumari Rani, Rajeev Reddy Pappuru, Savitri Sharma, Sisinthy Shivaji

**Affiliations:** 1 Smt. Kanuri Santhamma Centre for Vitreo-Retinal Diseases, L. V. Prasad Eye Institute, Kallam Anji Reddy Campus, Hyderabad, 500034 India; 2 Prof. Brien Holden Eye Research Centre, L. V. Prasad Eye Institute, Kallam Anji Reddy Campus, Hyderabad, 500034 India; 3 Internal Medicine, L. V. Prasad Eye Institute, Kallam Anji Reddy Campus, Hyderabad, 500034 India

**Keywords:** Microbial communities, Retinal diseases

## Abstract

Gut bacterial microbiome dysbiosis in type 2 Diabetes Mellitus (T2DM) has been reported, but such an association with Diabetic Retinopathy (DR) is not known. We explored possible link between gut bacterial microbiome dysbiosis and DR. Using fecal samples of healthy controls (HC) and people with T2DM with/without DR, gut bacterial communities were analysed using 16S rRNA gene sequencing and data analysed using QIIME and R software. Dysbiosis in the gut microbiomes, at phyla and genera level, was observed in people with T2DM and DR compared to HC. People with DR exhibited greater discrimination from HC. Microbiomes of people with T2DM and DR were also significantly different. Both DM and DR microbiomes showed a decrease in anti-inflammatory, probiotic and other bacteria that could be pathogenic, compared to HC, and the observed change was more pronounced in people with DR. This is the first report demonstrating dysbiosis in the gut microbiome (alteration in the diversity and abundance at the phyla and genera level) in people with DR compared to HC. Such studies would help in developing novel and targeted therapies to improve treatment of DR.

## Introduction

Diabetes mellitus (DM) is a multi-organ metabolic disorder and diabetic retinopathy (DR) is the most common blinding ophthalmic disorder in people with DM. The International Diabetes Federation (IDF) has estimated that DM currently affects over 463 million people in the world and this is expected to increase to 700 million in 2045^1^. DR is known to develop within 5 years of the onset of type 2 DM (T2DM), but this time is variable and not all DM patients develop DR. Does it suggest an inherent difference in the individuals in addition to the known metabolic factors? Dysbiosis, an alterations / imbalance in the gut microbiome may be associated with inter-individual differences and could thus drive DR. The human gut microbiome is represented by 1,500 different species^2,3^. Dysbiosis of the gut microbiome has been implicated in a variety of diseases including inflammatory diseases (obesity, inflammatory bowel disease), auto-immune diseases (rheumatoid arthritis, muscular dystrophy, DM), various types of cancers and mental disorders (Alzheimer’s disease, anxiety and autistic disorders)^4–9^. Others, and we have reported possible connections between gut microbiome dysbiosis and ocular diseases^10–12^, such as uveitis^13–17^, ocular mucosal disease^18^, bacterial and fungal keratitis^19,20^ and age-related macular degeneration^21,22^. Earlier studies have documented bacterial microbiome dysbiosis in people with DM^5–7^ and indicated that type 1 diabetes (T1D) onset is preceded by islet autoimmunity, dysregulation of lipid and amino acid metabolism^5^, increase in inflammation-associated organisms and pathways with higher levels of human β-defensin 2^23^. In a recent study, Tetz et al.^24^ demonstrated for the first time that amyloid-producing *E*. *coli*, their phages, and bacteria-derived amyloid might be involved in pro-diabetic pathway activation in children at risk for T1D. Earlier studies by Zhao et al.^25^ indicated that intestinal virome changes precede autoimmunity in T1D-susceptible children. We are unsure if gut bacterial microbiome dysbiosis is also reflected in people with DR and if the dysbiosis is similar in people with DM and DR.

The primary aim of the current study was to identify the gut microbiomes of people with DM and DR, compare these with the microbiomes of healthy individuals, and to assess whether microbiome dysbiosis is associated in people with DR. In this study DR patients were categorised into two subgroups namely Proliferative Diabetic retinopathy (PDR) and Non-Proliferative Diabetic Retinopathy (NPDR) (https://www.aoa.org/patients-and-public/eye-and-vision-problems/glossary-of-eye-and-vision-conditions/diabetic-retinopathy). NPDR individuals are in the early stage of the disease with blood vessels in the retina weakened, exhibiting tiny bulges called microaneurysms which may leak fluid into the retina leading to swelling of the macula. In contrast, in PDR the disease is more advanced, the retina is deprived of oxygen and new fragile blood vessels grow in the retina and also extend into the vitreous of the eye. The new blood vessels may also bleed into the vitreous, thus clouding vision. Microbiomes from both NPDR and PDR were analysed for dysbiotic changes and compared to T2DM and HC individuals. Such studies may lead to identification of specific bacterial associations in microbiomes in people with DR and help in developing novel therapies for treatment of DR.

## Results

The study population included people attending the eye care facility at the L V Prasad Eye Institute, Hyderabad, India. A total of 83 individuals were recruited as 3 distinct cohorts and included 30 healthy controls (HC) (17 males and 13 females; mean age 52.2 years, range 38–81 years), 25 people with T2DM without DR (14 males and 11 females; mean age 57.3 years, range 41–71 years), and 28 people with T2DM and DR (21 males and 7 females; mean age 55.2 years, range 44–69). The subjects were recruited from two adjacent states in South India (Telangana—90.36% and Andhra Pradesh—9.64%) (Table 1). All recruited individuals in HC, T2DM and DR were matched for age (*p* = 0.069), gender (*p* = 0.294) (Table 1), region of origin and all the confounding factors (except patient DR005 who had chronic kidney disease and DR015 and DR022 who had diabetic nephropathy), listed in Methods thus implying that our comparisons between the cohorts would be reliable.

**Table 1:** Demographic and clinical characteristics of healthy controls (HC), type 2 diabetes mellitus (T2DM) and diabetic retinopathy (DR) individuals.

Variable	HC	T2DM	DR
Sample Size	30	25	28
Mean age* (years)	52.2	57.3	55.07
Age range (years)	38–81	41–71	44–69
Gender* M: F	17: 13	14: 11	21: 7
**Region**			
Telangana	30	22	23
Andhra Pradesh	0	3	5
Diet*			
Non veg	23	24	24
Veg	7	1	4
Type 2 DM	0	†New DM : 15 ‡Known DM: 10	†New DM: 0 ‡Known DM: 28
Hypertension	0	11	20
Diabetic Retinopathy	NPDR: 0 PDR: 0	NPDR: 0 PDR: 0	NPDR: 9 PDR: 19
DM medication^+++^	0	25	28
Anti-hypertension medication	0	11	20

*Indicates *p* > 0.05—not significant.

^†^New-DMs—patients diagnosed as T2DM recently and taking anti-diabetes medication for the last 4 weeks.

^‡^Known-DMs—patients with T2DM and taking anti-diabetes medication for the last 1 year.

NPDR—Non-Proliferative Diabetic Retinopathy; PDR—Proliferative Diabetic Retinopathy.

^+++^Metformin or combinations of Metformin and/or Insulin.

### Rarefaction analysis and diversity indices

**Figure 1:**
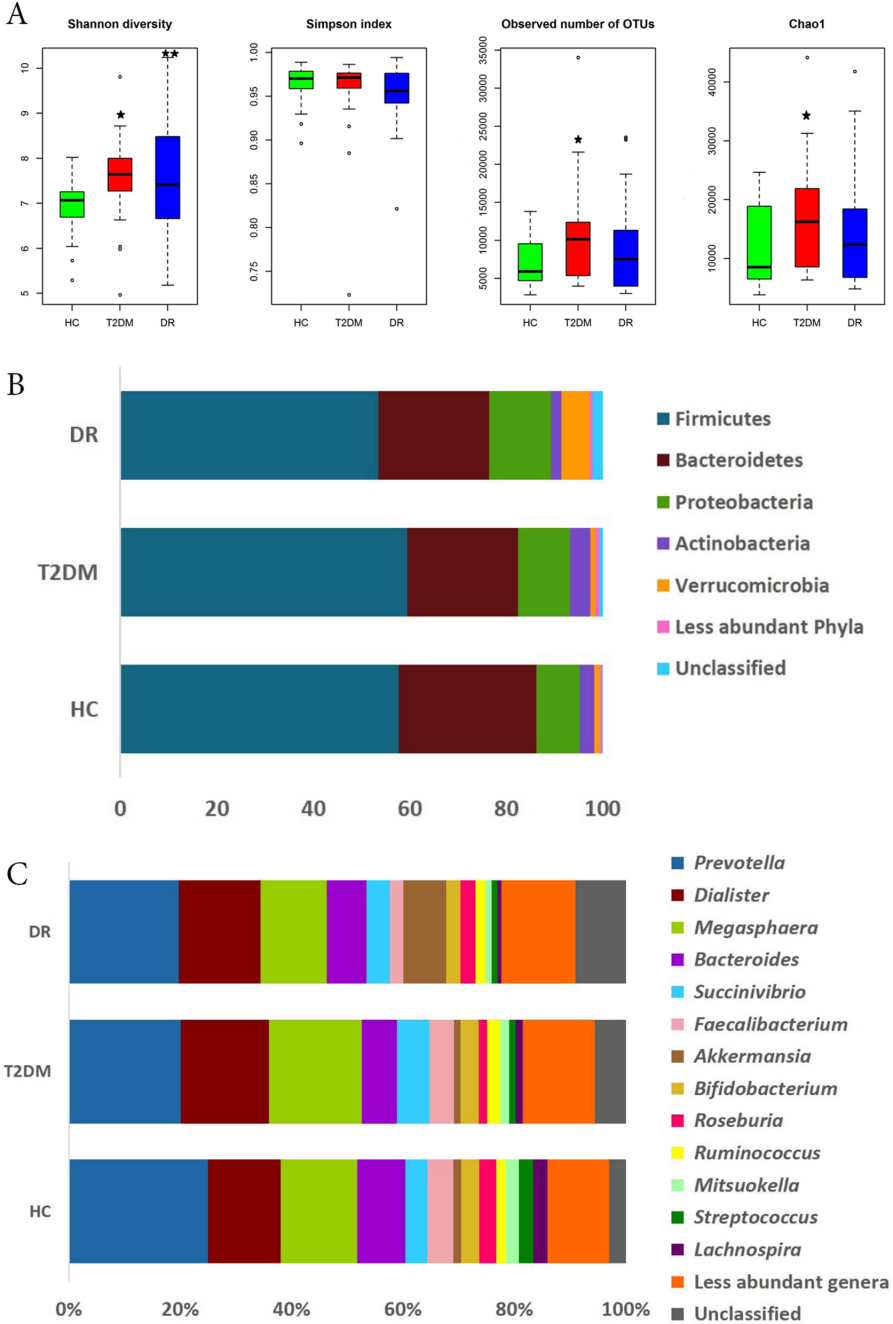
Bacterial diversity in the gut microbiomes of healthy controls (HC, n = 30), Type 2 Diabetes mellitus (T2DM, n = 24) and Diabetic Retinopathy (DR, n = 28) individuals. (**A**) Alpha diversity indices (Shannon diversity index, Simpson index (evenness), number of observed OTUs, and Chao1 index (richness), (**B**) average abundance of different bacterial phyla and (**C**) average abundance of different bacterial genera. ★ indicates significant difference between HC and T2DM and ★★ indicates significant difference between HC and DR by Student’s t-test (*p* < 0.05). Figure 1A was generated using R software version 3.4.3. R: A language and environment for statistical computing (http://www.R-project.org/).

A total of 27,783,944 high quality reads (HQ) (chimeric sequences removed and with a mean Phred score less than 25), were generated from the fecal bacterial microbiomes of 30 HC, 24 T2DM and 28 DR individuals. One T2DM sample (DM007) was excluded since the patient had phthisis bulbi, uveal coloboma and rhegmatogenous retinal detachment. The average number of HQ reads per microbiome was 338,829; the average reads followed the sequence of HC (416,370 reads) > T2DM (333,464 reads) > DR (260,346 reads) per microbiome. The difference between the reads of the individual samples across the three cohorts was not statistically significant (*p* = 0.806). Rarefaction curves of the gut microbiomes of all individuals showed a tendency to plateau, indicating reasonable sequencing depth and coverage for the sequenced samples (Supplementary Fig. S1). Alpha diversity indices namely Shannon diversity index, number of observed OTUs, and Chao1 index (richness) were significantly different between the bacterial gut microbiomes of HC and T2DM. Shannon diversity index alone was significantly different between HC and DR individuals (*p* < 0.05), whereas none of the indices were significantly different between T2DM and DR (Fig. 1A).

### Bacterial communities inhabiting guts of HC, T2DM and DR individuals

The average number of HQ reads in the gut microbiomes that could be assigned to an OTU with at least two reads per OTU was 86.41% reads (at 97% sequence identity). The remaining 13.59% reads were singletons (only one read is assigned to an OTU) which have not been considered. A total of 3539 OTUs were identified from the three cohorts consisting of 1737 reference OTUs and 1802 denovo OTUs (Supplementary Table S1). Fifteen phyla were identified in the gut microbiomes of HC, T2DM and DR individuals (Table 2, Supplementary Fig. S2, Fig. 1B). Firmicutes, Bacteroidetes, Proteobacteria, Actinobacteria, Verrucomicrobia, Cyanobacteria, Elusimicrobia, Tenericutes, Euryarchaeota, TM7, Lentisphaerae and Synergistetes were present in all the 82 microbiomes. Four phyla, namely Firmicutes, Bacteroidetes, Proteobacteria and Actinobacteria, were the most predominant with a combined average abundance of 98.31%, 97.46% and 91.47% in HC, T2DM and DR respectively. The remaining 11 minor phyla together constituted a mean abundance of 1.69% in HC, 2.53% in T2DM and 8.53% in DR. None of the major phyla exhibited any difference at the abundance level between HC and T2DM, but 8 minor phyla varied significantly between the two cohorts (Table 2). In contrast, two major phyla, Bacteroidetes and Actinobacteria were significantly less abundant in the gut microbiome of people with DR compared to HC (*p* < 0.05). The abundance of Actinobacteria was also significantly reduced in DR patients compared to T2DM. Among the 11 minor phyla, 10 were significantly different across all three cohorts (Table 2).

**Table 2:** Mean abundance (%) of bacterial phyla in the gut microbiomes of healthy controls (HC, n = 30), type 2 diabetes mellitus (T2DM, n = 24) and diabetic retinopathy (DR, n = 28) individuals.

S. No	Phyla	HC (n = 30)			T2DM (n = 24)			DR (n = 28)			*p* value			
		Mean	Range	Density	Mean	Range	Density	Mean	Range	Density	HC vs. T2DM vs. DR	HC vs. T2DM	HC vs. DR	T2DM vs. DR
1	Firmicutes	57.72	38.96–77.31	30	59.42	31.85–85.35	24	53.44	17.53–76.01	28	0.459	0.712	0.447	0.26
2	Bacteroidetes	28.58	6.24–43.83	30	23.09	4.41–38.64	24	22.95	7.43–51.98	28	0.065	0.128	**0.032**	0.75
3	Proteobacteria	8.94	2.7–21.96	30	10.73	1.29–32.47	24	12.76	2.07–46.03	28	0.71	0.924	0.454	0.668
4	Actinobacteria	3.07	0.25–12.8	30	4.22	0.78–18.93	24	2.32	0.5–24.45	28	**0.003**	0.415	**0.021**	**0**
Average abundance		98.31			97.46			91.47						
5	Verrucomicrobia	1.04	0.06–16.87	30	0.9	0–10.99	24	5.69	1.01–54.31	28	**0**	0.678	**0**	**0**
6	Cyanobacteria	0.3	0.02–2	30	0.24	0.02–1.61	24	0.19	0.02–1.92	28	0.465	0.924	0.638	0.124
7	Elusimicrobia	0.11	0.01–1.98	30	0.01	0–0.08	24	0.04	0.01–0.55	28	**0**	**0**	0.197	**0**
8	Tenericutes	0.05	0.01–0.21	30	0.08	0–0.38	24	0.17	0.02–1.29	28	**0.032**	0.141	**0.011**	0.663
9	Euryarchaeota	0.03	0.01–0.23	30	0.41	0.04–3.63	24	0.16	0–1.36	28	**0**	**0**	0.163	**0**
10	Fusobacteria	0.03	0–0.19	27	0.01	0–0.12	24	0	0–0.02	28	**0**	**0.002**	**0**	**0**
11	TM7	0.02	0–0.17	30	0	0–0.01	24	0	0–0.02	28	**0**	**0**	**0**	0.75
12	Crenarchaeota	0.01	0–0.06	20	0	0–0	0	0	0–0	0	**0**	**0**	**0**	NA
13	Spirochaetes	0.01	0–0.04	22	0.17	0–2.16	20	0	0–0	19	**0**	**0**	**0**	**0**
14	Lentisphaerae	0	0–0.02	30	0.02	0–0.2	24	0.01	0–0.06	28	**0**	**0**	**0.006**	**0.017**
15	Synergistetes	0	0–0.01	30	0.01	0–0.04	24	0.04	0.01–0.63	28	**0**	**0**	**0**	**0**
16	Unclassified	0.09	0.03–0.24	30	0.68	0.39–2.75	24	2.23	0.45–12.93	28	**0**	**0**	**0**	**0**
Average abundance		1.69			2.53			8.53						

**Figure 2:**
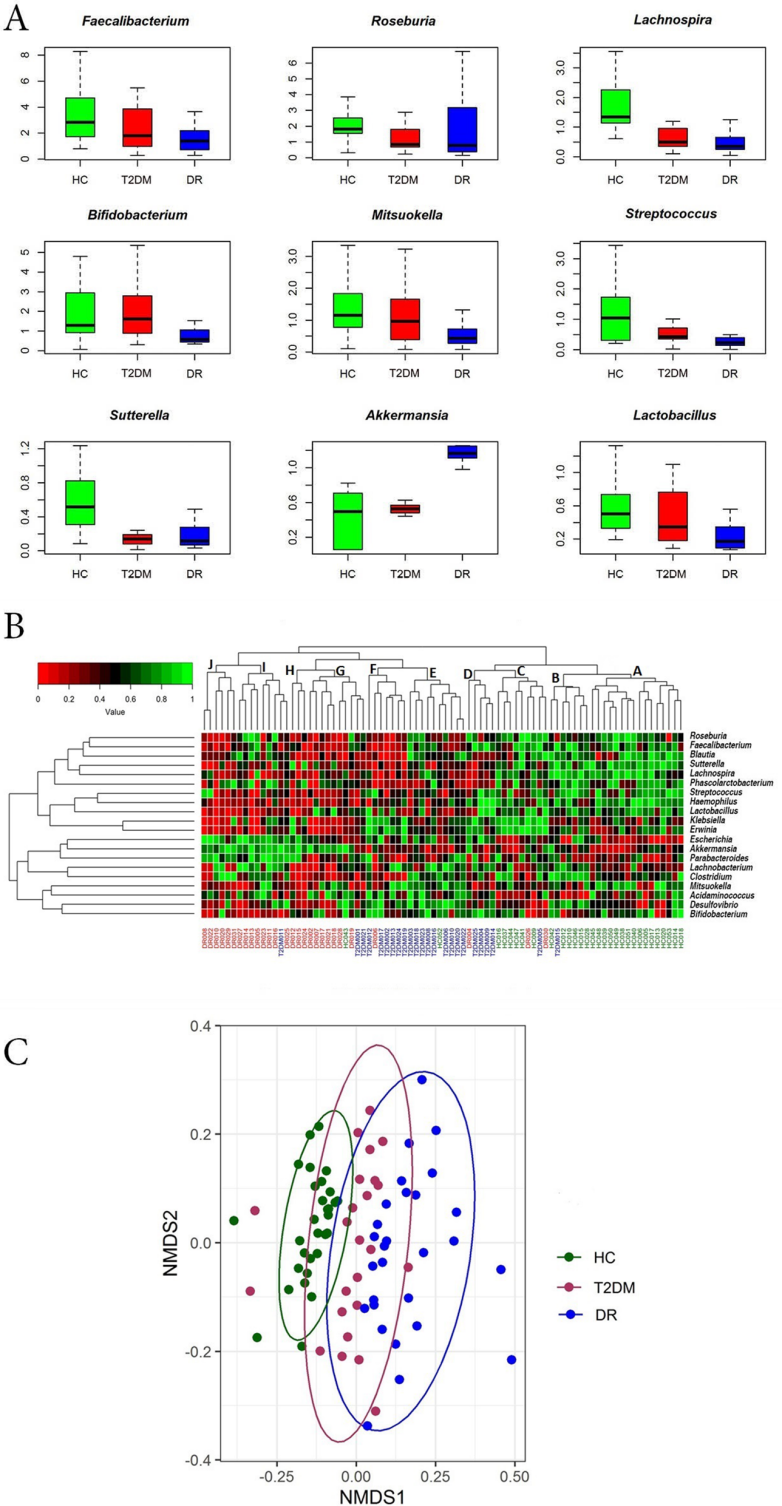
Gut microbiomes differ significantly across (HC, n = 30), Type 2 Diabetes mellitus (T2DM, n = 24) and Diabetic Retinopathy (DR, n = 28) individuals. (**A**) Bacterial genera exhibiting significant (Kruskal Wallis test, BH corrected *p* < 0.05) differential abundance in the gut microbiomes from HC, T2DM and DR individuals. Differentially abundant genera having a median abundance of > 0.5% in at least one group of samples have been depicted. Median abundances (horizontal line) and interquartile ranges have been indicated in the plots. (**B**) Two dimensional heat map showing rank normalized abundances determined by Kruskal Wallis test (scaled between 0 and 1) of 20 differentially abundant bacterial genera in gut microbiomes from HC, T2DM and DR individuals. The discriminating genera have been arranged along the two dimensions (axes) based on hierarchical clustering. **(C)** Beta diversity analysis using NMDS plots based on Bray–Curtis dissimilarity of bacterial OTUs in the gut microbiomes of HC, T2DM and DR. The bacterial community appeared to vary significantly across HC, T2DM and DR (PERMANOVA, *p* = 0.001). Figures were generated using R software version 3.4.3. R: A language and environment for statistical computing (http://www.R-project.org/). Packages matrixStats v.0.55.0 (https://cran.r-project.org/web/packages/matrixStats/index.html) and gplots v. 3.0.4 (https://cran.r-project.org/web/packages/gplots/index.html) were used to generate the heatmap in Fig. 2B. Packages ggplot2 v.3.2.1 (https://cran.r-project.org/web/packages/ggplot2/index.html), vegan v.2.5-6 (https://cran.r-project.org/web/packages/vegan/index.html) and rgl v. 0.100.54 (https://cran.r-project.org/web/packages/rgl/index.html) were used to generate the NMDS plot in Fig. 2C.

In the gut microbiomes of HC, T2DM and DR, 93 bacterial genera were identified out of which 49 genera were shared between the three cohorts (Supplementary Table S2, Supplementary Fig. S3, Fig. 1C). Compared to the abundance in HC, several genera were significantly decreased in the bacterial microbiomes of people with T2DM (10 genera) and DR (20 genera) (Tables 3 and 4) and 7 genera were common to people with both T2DM and DR. In addition, compared to HC the abundance of 8 and 11 genera were significantly increased in T2DM and DR patients respectively and 3 genera (*Acidaminococcus, Escherichia* and *Enterobacter*) were common to both (Tables 3 and 4). We also observed that several genera either decreased (13 genera) or increased (6 genera) in DR patients compared to T2DM patients (Table 5). The highly abundant discriminatory genera between the three cohorts are shown in Fig. 2A.

**Table 3:** Bacterial genera exhibiting significant differential abundance (BH corrected *p* < 0.05) between the gut microbiomes of healthy controls (HC, n = 30) and type 2 diabetes mellitus (T2DM, n = 24) individuals.

S. no.	Genus	Median abundance (%)		Characteristics	References
		HC	T2DM		
**Genera decreased in T2DM**					
1	*Roseburia*	1.82	0.85	^a^Anti-inflammatory	^65,66^
2	*Lachnospira*	1.34	0.5	^a^Anti-inflammatory	^65^
3	*Sutterella*	0.52	0.14	Pro-inflammatory	^67^
4	*Coprococcus*	0.37	0.21	^a^Anti-inflammatory	^65,66^
5	*Phascolarctobacterium*	0.23	0.02	^a^Anti-inflammatory	^68,69^
6	*Haemophilus*	0.17	0.06	Pathogen	^70^
7	*Blautia*	0.15	0.08	^a^Anti-inflammatory/antibacterial	^65,69,71,72^
8	*Comamonas*	0.01	0	Pathogen	^73^
9	*Anaerostipes*	0.01	0	^a^Anti-inflammatory	^66,71^
10	Turicibacter	0.01	0	Not known	
**Genera increased in T2DM**					
1	*Acidaminococcus*	0.09	0.21	^a^Anti-inflammatory	^65,68^
2	*Escherichia*	0.04	0.13	Pathogen	^74^
3	*Lachnobacterium*	0.04	0.09	Not known	
4	*Butyricimonas*	0.01	0.09	^a^Anti-inflammatory	^65^
5	*Enterobacter*	0.01	0.07	Pathogen	^74^
6	*Methanobrevibacter*	0.01	0.05	Pro-inflammatory	^75^
7	*Treponema*	0	0.03	Pathogen	^76^
8	*Weissella*	0	0.01	Probiotic/anti-inflammatory/antibacterial	^77,78^

^a^Based on the capability to produce short chain fatty acid.

**Table 4:** Bacterial genera exhibiting significant differential abundance (BH corrected *p* < 0.05) between the gut microbiomes of healthy controls (HC, n = 30) and diabetic retinopathy (DR, n = 28) individuals.

S. n.o	Genus	Median Abundance (%)		Characteristics	References
		HC	DR		
**Genera decreased in DR**					
1	*Faecalibacterium*	2.84	1.39	^a^Anti-inflammatory	^65,66,68^
2	*Roseburia*	1.82	0.8	^a^Anti-inflammatory	^65,66^
3	*Lachnospira*	1.34	0.36	^a^Anti-inflammatory	^65^
4	*Bifidobacterium*	1.29	0.58	Probiotic/^a^anti-inflammatory/antibacterial	^66,68,79,80^
5	*Mitsuokella*	1.15	0.43	^a^Anti-inflammatory	^81^
6	*Streptococcus*	1.05	0.23	Probiotic/^a^anti-inflammatory/pathogen	^66,79,82^
7	*Sutterella*	0.52	0.12	Pro-inflammatory	^67^
8	*Lactobacillus*	0.5	0.17	Probiotic/^a^anti-inflammatory/antibacterial	^66,79,83,84^
9	*Clostridium*	0.17	0.11	Pathogen/^a^anti-inflammatory	^65,66,81,85^
10	*Haemophilus*	0.17	0.01	Pathogen	^70^
11	*Blautia*	0.15	0.08	^a^Anti-inflammatory/antibacterial	^65,69,71,72^
12	*Erwinia*	0.12	0.01	Pathogen	^86^
13	*Desulfovibrio*	0.06	0.04	Pathogen	^87^
14	*Bulleidia*	0.02	0	Pathogen	^88^
15	*Butyrivibrio*	0.02	0	^a^Anti-inflammatory	^65,81^
16	*Asteroleplasma*	0.014	0.01	Not known	
17	*Anaerovibrio*	0.01	0	Not known	
18	*Comamonas*	0.01	0	Pathogen	^73^
19	*Rothia*	0.01	0	Pathogen	^89^
20	*Turicibacter*	0.01	0	Not known	
**Genera increased in DR**					
1	*Akkermansia*	0.5	1.16	^a^Anti-inflammatory	^66^
2	*Parabacteroides*	0.2	0.45	^a^Anti-inflammatory	^69,71^
3	*Megamonas*	0.14	0.27	^a^Anti-inflammatory	^69^
4	*Acidaminococcus*	0.09	0.22	^a^Anti-inflammatory	^65,68^
5	*Escherichia*	0.04	0.2	Pathogen	^74^
6	*Alistipes*	0.01	0.03	^a^Anti-inflammatory	^69,71^
7	*Enterobacter*	0.01	0.03	Pathogen	^74^
8	*Cloacibacillus*	0	0.03	Pathogen	^90^
9	*Enterococcus*	0	0.02	Pathogen/probiotic	^84,91^
10	*Oxalobacter*	0	0.01	Probiotic	^92^
11	*Shigella*	0	0.01	Pathogen/pro-inflammatory	^93^

^a^Based on the capability to produce short chain fatty acid.

**Table 5:** Bacterial genera exhibiting significant differential abundance (BH corrected *p* < 0.05) between the gut microbiomes of type 2 diabetes mellitus (T2DM, n = 24) and diabetic retinopathy (DR, n = 28) individuals.

S. no.	Genus	Median abundance (%)		Characteristics	References
		T2DM	DR		
**Genera decreased in DR**					
1	*Bifidobacterium*	1.62	0.58	Probiotic/^a^anti-inflammatory/antibacterial	^66,68,79,80^
2	*Mitsuokella*	0.97	0.43	^a^Anti-inflammatory	^81^
3	*Streptococcus*	0.43	0.23	Probiotic/^a^anti-inflammatory/pathogen	^66,79,82^
4	*Klebsiella*	0.31	0.06	Pathogen	^74^
5	*Desulfovibrio*	0.16	0.04	Pathogen	^87^
6	*Lachnobacterium*	0.13	0.03	Not known	
7	*Erwinia*	0.09	0.01	Pathogen	^86^
8	*Treponema*	0.09	0	Pathogen	^76^
9	*Methanobrevibacter*	0.07	0.03	Pro-inflammatory	^75^
10	*Haemophilus*	0.06	0.01	Pathogen	^70^
11	*Asteroleplasma*	0.05	0.01	Not known	
12	*Anaerovibrio*	0.01	0	Not known	
13	*Weissella*	0.01	0	Probiotic/anti-inflammatory/antibacterial	^77,78^
**Genera increased in DR**					
1	*Akkermansia*	0.53	1.16	^a^Anti-inflammatory	^66^
2	*Phascolarctobacterium*	0.02	0.14	^a^Anti-inflammatory	^68,69^
3	*Alistipes*	0.01	0.03	^a^Anti-inflammatory	^69,71^
4	*Shigella*	0.01	0.03	Pathogen/Pro-inflammatory	^93^
5	*Cloacibacillus*	0	0.02	Pathogen	^90^
6	*Enterococcus*	0	0.01	Pathogen/probiotic	^84,91^

^a^Based on the capability to produce short chain fatty acid.

We also categorised T2DM patients into two subgroups, namely ‘new’—T2DM (diagnosed as T2DM and on anti-diabetes medication for the last 4 weeks, n = 14) and ‘known’—T2DM (patients with T2DM and taking anti-diabetes medication for the last 1 year, n = 10) and DR into two subgroups namely ‘PDR’ (Proliferative Diabetic retinopathy, n = 19) and ‘NPDR’ (Non-Proliferative Diabetic Retinopathy, n = 9). Wilcoxon test did not identify any discriminatory genera between the subgroups of T2DM and DR implying that the microbiomes were similar and age of T2DM and severity of DR did not influence the results.

A two-dimensional heatmap analysis (Fig. 2B) of the 20 differentially abundant bacterial genera in the gut microbiomes indicated a clear separation of HC, T2DM and DR microbiomes. The majority of the HC (28 of 30) and T2DM (19 of 24) microbiomes clustered together into 3 sub-clades each (sub-clades A, B and C in HC and D, E and F in T2DM) whereas 24 of 28 DR microbiomes clustered into 4 sub-clades (sub-clades G, H, I and J) (Fig. 2B). Further, in agreement with the heatmap analysis, the β (Beta) diversity of HC, T2DM and DR microbiomes analysed by NMDS plots using Bray–Curtis dissimilarity of OTUs and discriminating genera, distinguished the three cohorts into 3 clusters (*p* = 0.001) (Fig. 2C and Supplementary Fig. S4).

### Functional profile of gut bacterial communities of HC, T2DM and DR patients

KEGG functional pathway analysis predicted significant differences in the HC, T2DM and DR bacterial microbiomes. Compared to HC samples, 17 pathways were significantly enriched and 16 pathways were significantly reduced in T2DM samples (Supplementary Table S3) whereas in DR samples, an increase in 60 pathways and decrease in 34 pathways were observed (Supplementary Table S4). When T2DM and DR functional pathways were compared, we observed enhancement of 29 pathways and reduction of 10 pathways in DR samples (Supplementary Table S5).

### Interactions between the bacterial genera in the gut of healthy controls and people with T2DM and DR

**Figure 3:**
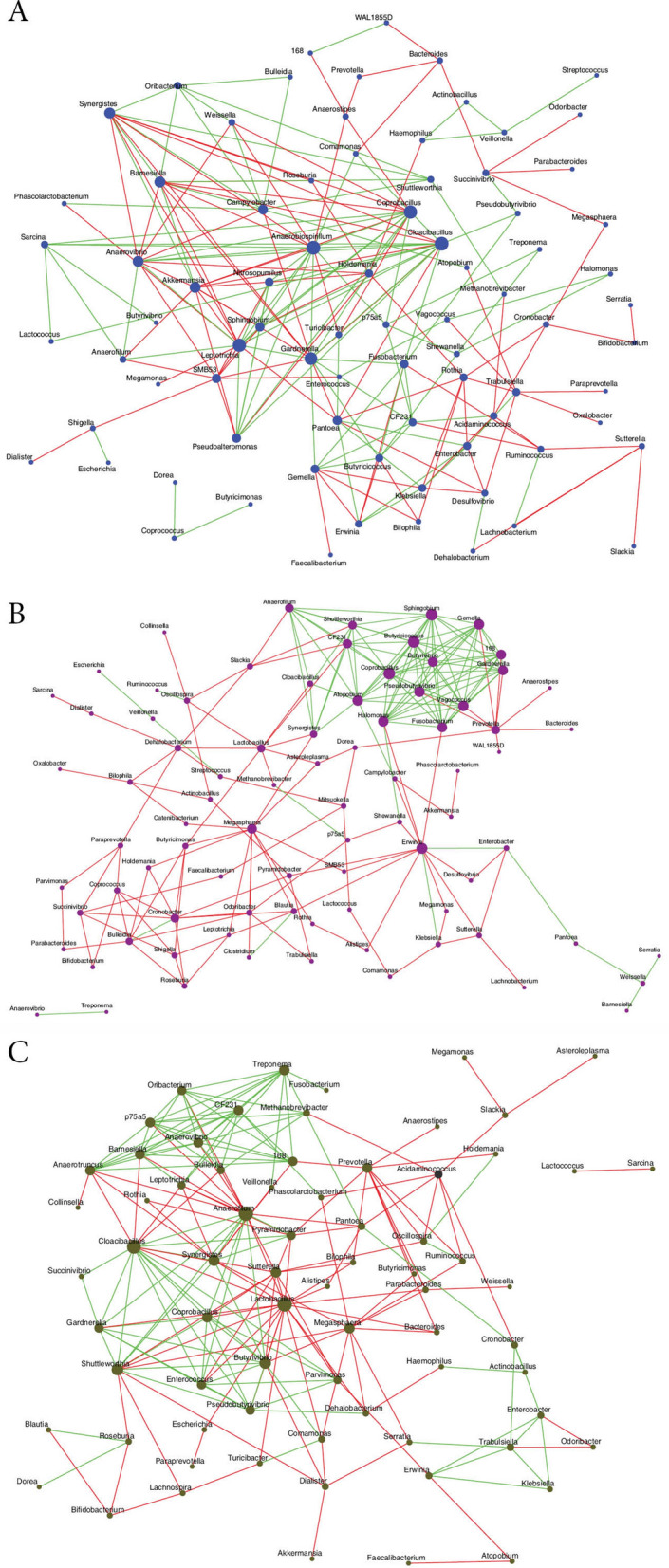
Significant co-occurrence and co-exclusion relationships at genus level in the gut microbiomes of healthy controls (HC, n = 30), Type 2 Diabetes mellitus (T2DM, n = 24) and Diabetic Retinopathy (DR, n = 28) individuals. Interaction of bacterial genera in the gut microbiomes of healthy controls (**A**), Type 2 Diabetes mellitus (**B**) and Diabetic retinopathy (**C**) individuals (based on correlation of genera-level abundance). The node sizes in the network correspond to their degree of interaction. The positive and negative correlations / interactions are indicated with green and red edges respectively.

Three interaction networks were generated for the gut microbiomes of HC, T2DM and DR based on pair-wise correlations between abundances of different microbial genera. A single well-connected network was observed in all the 3 cohorts (Fig. 3) and several ‘hub’ genera or ‘nodes’ (with high degree of interaction > 10) could be identified. These hub genera interacted either positively or negatively or both positively and negatively with other genera. The number of hub genera followed the sequence of T2DM (n = 14) > HC (n = 9) = DR (n = 9). Among the 9 hub genera, HC microbiomes shared 2 (*Coprobacillus* and *Gardnerella*) with T2DM and 2 (*Cloacibacillus* and *Synergistes*) with DR; the remaining 5 (*Akkermansia, Anaerobiospirillum, Anaerovibrio, Barnesiella* and *Leptotrichia*) were unique to HC. T2DM and DR microbiomes shared 2 hub genera (*Butyrivibrio* and *Megasphaera*). The genera *Atopobium, Butyricicoccus, Erwinia, Fusobacterium, Gemella, Halomonas, Pseudobutyrivibrio, 1–68* (*Tissierellaceae*), *Vagococcus* and *Sphingobium* were found to be the unique hubs in T2DM microbiomes. In DR, *Anaerofilum, Lactobacillus, Shuttleworthia, Sutterella* and *Treponema* were the unique hubs. Thus, it was apparent that all the 3 interaction networks were distinctly different.

## Discussion

The major resident microbes in the gut of people with T2DM are affiliated to the phyla *Firmicutes*, *Bacteroidete*s, *Proteobacteria* and *Actinobacteria*. The abundance of these four phyla in T2DM was not significantly different from HC. This is in contrast to earlier studies which showed reports indicating either increase^26^ or decrease^27^ in abundance of *Firmicutes* in T2DM compared to HC. Pandolfi et al.^28^ suggested association of *Firmicutes* with obesity and that diabetes is probably mediated by insulin resistance which is a common attribute of both the conditions. Several earlier studies^6,29–34^ have indicated that 13 genera were either increased or decreased in people with DM (Supplementary Table S6). *Bacteroides* was the most prominent genera in most of the studies. In the present study, we detected 11 of these 13 genera (except *Eggerthellalenta* and *Eubacterium*) in HC and in people with T2DM (Supplementary Table S2). In addition, we observed significant decrease in 10 (*Roseburia*, *Lachnospira, Sutterella, Coprococcus, Phascolarctobacterium, Haemophilus, Blautia, Comamonas, Anaerostipes* and *Turicibacter*) and increase in 8 other genera in T2DM compared to HC (Table 3). This may imply that these 11 genera are specific to the Indians with T2DM. However, the similarity was less when compared to another study on Indian subjects from Pune, by Bhute et al.^26^. They analysed the bacterial microbiomes of healthy controls (HC), new DMs (NDM) and known DMs (KDM) and observed that 20 OTUs were highly discriminative among the three groups and it included 11 taxa (*Oscillospira, Faecalibacterium prausnitzii, Bifidobacterium, Bifidobacterium adolescentis, Prevotella copri, Lachnospiraceae, Lactobacillus ruminis, Ruminococcaceae, Roseburia, Collinsella aerofaciens* and *Streptocoocus*)*.* All the 11 discriminating taxa of Bhute et al.^26^ were identified by us but only two of the taxa *Roseburia* and *Lachnospira*, were significantly reduced in abundance in T2DM. Members of families Lachnospiraceae like *Lachnospira* produce short chain fatty acid (SCFA), which confer health benefits. Thus, an increase in abundance in *Lachnospira* in HC would support a healthy state and the observed decrease in T2DM may have a role in diabetes^35^. While it is difficult to interpret the observed discrepancy between this and Bhute et al.^26^ study, we wonder if the difference in the region of study (south central India, Hyderabad versus central India, Pune) and food habit (predominantly non-vegetarian in south central India and predominantly vegetarian in central India) has any influence.

Considering that T2DM is like an inflammatory disease^36^ it would not be surprising to record an increase in pro-inflammatory bacteria and decrease in anti-inflammatory bacteria in T2DM. Accordingly, we observed that the anti-inflammatory bacteria (*Roseburia, Lachnospira, Coprococcus, Phascolarctobacterium, Blautia* and *Anaerostipes*)^37–41^ were decreased whereas the pro-inflammatory bacteria (*Escherichia, Enterobacter, Methanobrevibacter* and *Treponema)*^41,42^ were increased in abundance in T2DM compared to HC (Table 3). An increase in a few pro-inflammatory and a few anti-inflammatory bacteria were also seen in HC and T2DM respectively (Table 3). Results imply that a balance between the anti- and pro-inflammatory bacteria is crucial to HC, but evidently there has to be a dominance of anti-inflammatory over pro-inflammatory bacteria. That the microbiomes are different between HC and T2DM is also obvious from the two dimensional heat map and β-diversity analysis (PERMANOVA, *p* = 0.001). Thus, segregation of HC and T2DM is robust and is a reflection of the compositional differences in the bacterial communities.

Metformin is normally used by all T2DM patients. The drug is known to induce dysbiotic changes in the gut microbiota. It has been demonstrated that metformin decreased the Shannon index (α-diversity)^43,44^ and reshaped the gut microbiome^44,45^ which could be attributed to metformin induced decrease in the abundance of several genera (taxa) like *Intestinibacter*, *Clostridium*, *Terrisporobacter*, *Senegalimassilia, Bacteroides fragilis* and Lachnospiraceae and increase in the abundance of Enterobacteriaceae, *Staphylococcus, Escherichia*/*Shigella, Bilophila, Lachnoclostridium*, *Caproiciproducens,* *Tyzzerella* and *Prevotella* in T2DM individuals^27,32,44–49^. But, the taxa identified as showing abundance changes due to metformin have been inconsistent across the studies. For instance, none of the above genera showed any significant difference in the study by Elbere et al.^43^ who indicated that four additional taxa namely *Peptostreptococcaceae*, Clostridiaceae unclassified, *Asaccharospora* and *Romboutsia* were decreased. In a few studies, relative abundance of *Escherichia/Shigella* genus did not exhibit significant changes on metformin treatment^43,49^. Abundance changes in certain bacteria with beneficial effects like increase in *Akkermansia muciniphila,* a mucin degrading bacteria^45,49^ is less clear^32,43,47,50^ so also decrease in *B. fragilis*^44^ which regulates bile acids^44^ is not consistent. The data on metformin induced changes in abundance in butyrate producers (*Roseburia, Subdoligranulum, Faecalibacterium*) was also inconsistent^6,32,47^. In the present study, all the T2DM individuals were under metformin treatment and the gut microbiomes of T2DM individuals could be discriminated from HC microbiomes by Heatmap and NMDS plots and significant differences were observed in abundance of genera with decrease in 10 and increase in 8 genera compared to healthy individuals (Table 3). Significant decrease in *Lachnospira* and increase in *Enterobacter* and *Escherichia* were the only two similarities seen with this study and that of the above studies. These observations confirm that abundance changes in taxa due to metformin are inconsistent across studies. Our results may also imply that all the other differences at the genera level between HC and T2DM observed in this study may be more associated with T2DM.

The observed increase in *Escherichia* is relevant to diabetes development since amyloid-producing *E. coli* and their phages are associated with autoimmunity and protein misfolding, considered as one of the possible pathological pathways of diabetes disease progression^24^ and other diseases. Chen et al.^51^ demonstrated that in rodents and nematodes bacterial amyloid proteins influence neurodegenerative processes by misfolding of amyloid proteins such as alpha-synuclein. T2DM patients with DR represent a group that is less well managed (ill controlled blood sugar) and are also probably in the advanced stage of the disease. A recent study has indicated reduction in severity of DR in diabetic mice following gut microbiome restructuring by altering the feeding patterns^52^. Yet, there is no evidence pointing at a “direct” role of gut dysbiosis in DR. The current study shows that the gut microbiomes in people with DR differed from HC and T2DM in relative abundance of phyla (Table 2) and several genera (Tables 4 and 5) and grouped separately in the heatmap (Fig. 2B) and in the NMDS plots (Fig. 2C and Supplementary Fig. S4). In the absence of studies on the human gut microbiome of people with DR we compared our results with a mouse model of diabetes (db/db mice), which exhibits characteristic features of DR such as impaired intestinal barrier function, increase in number of acellular capillaries and increase in retinal levels of inflammatory cells^44^. In this study of Beli et al.^52^, the most dominant phyla in the fecal bacterial microbiome of diabetic (db/db) mice were *Bacteroidetes*, *Firmicutes*, *Verrucomicrobia*, *Tenericutes*, *Actinobacteria* and *Proteobacteria* and the DR mice did not show increase in *Bacteroidetes* to *Firmicutes* ratio. Our observations in human samples were similar at the phyla level but differed in that we noted a decrease in the abundance of 20 genera and increases in 11 genera in people with DR compared to HC (Table 4).The difference in gut microbiomes between human and murine DR is least surprising since only 4% of the bacterial genes are known to share considerable identity between man and mouse^53^. In the present study, the microbiomes of three DR patients (DR005, DR015 and DR022) with kidney disease were also analysed and it was observed that the microbiomes were similar to other DR microbiomes both in the Heatmap analysis (Fig. 2B) and NMDS plots (Fig. 2C and Supplementary Fig. S4) implying that there is no significant difference in the gut bacterial community of DR patients with and without kidney disease.

Chronic inflammation is a prerequisite for the onset of DR and this may be mediated by the gut microbiota like *Akkermansia muciniphila, Bacteroidetes thetaiotaomicron* and *Escherichia coli* which enhance gut permeability and endotoxemia^54^. Production of SCFA, like butyrate is essential for gut integrity^55^. This is normally accomplished by butyrate-producing bacteria such as *Eubacterium*, *Fusobacterium*, *Anaerostipes*, *Roseburia*, and *Faecalibacterium*. These SCFA producing bacteria have anti-inflammatory effects^56^, reduce bacterial translocation across the intestine, maintain gut integrity^57^ and are associated with β-cell autoimmunity and insulin resistance^58^. Hence it is not surprising that the gut microbiota of healthy people without DM had several SCFA producing microorganisms and this is reduced both in the gut microbiomes of people with DM and DR. In our study, anti-inflammatory gut microbiota (*Roseburia, Lachnospira, Coprococcus, Phascolarctobacterium*, *Blautia* and *Anaerostipes*) were decreased in people with T2DM. In DR, in addition to the genera such as *Roseburia, Lachnospira* and *Blautia* several other anti-inflammatory genera like *Faecalibacterium, Bifidobacterium, Ruminococcus, Mitsuokella, Streptocoocus, lactobacillus* and *Butyrivibrio* were also decreased. Incidentally, we also observed that the pro-inflammatory bacterium *Sutterella,* many possibly pathogenic bacteria (*Clostridium*, *Haemophilus, Erwinia, Desulfovibrio, Bulleida, Rothia,* and *Comamonas*) and probiotic bacterium *Lactobacillus* were decreased in people with DR compared to HC and T2DM (Tables 4 and 5). In earlier studies of keratitis (bacterial and fungal) and uveitis (idiopathic and autoimmune), we have reported increase in pro-inflammatory and pathogenic organisms^15,16,19,20^. The opposite trend was seen in people with DR in the current study. Thus it might be prudent to infer that enhanced inflammation in people with DR is ascribed to decrease in anti-inflammatory bacteria rather than an increase in pro-inflammatory bacteria. In fact we observed that only one pro-inflammatory bacterium, *Shigella*, increased in DR compared to HC and also T2DM. It is still difficult to explain the observed decrease in possibly pathogenic bacteria in people with DR compared to people with DM and human controls. Along with increase or decrease in anti-inflammatory and/or possibly pathogenic bacteria in people with DR, we also noted a decrease in 2 probiotic bacteria namely *Bifidobacterium* and *Lactobacillus*. Thus it would appear that the altered balance between the pro-and anti-inflammatory gut microbiome and the presence of pathogenic organism could be influencing the status of DR.

Comparison of the three interaction networks of HC, T2DM and DR indicated a predominance of positive correlation in HC network compared to T2DM and DR. Two (*Akkermansia* and *Barnesiella*) of the nine hub genera in HC possessed anti-inflammatory properties and negatively interacted with four pathogenic hubs, *Anaerobiospirillum, Gardnerella*, *Cloacibacillus* and *Leptotrichia*. Such an interaction would be beneficial to HC since pathogenic genera could be neutralized by anti-inflammatory genera. In contrast, 14 hub genera were recognised in T2DM, which negatively interacted with other genera. In addition, an interesting feature of the T2DM network was that 12 of 14 hub genera positively interacted with one another and 6 of these 12 hub genera were pathogens (*Gardnerella, Atopobium, Fusobacterium, Gemella, Halomonas* and *Vagococcus*) and would support the concept that T2DM is a chronic inflammation. We also noted that anti-inflammatory hub genera, namely *Butyrivibrio*, *Butyricicoccus* and *Pseudobutyrivibrio* interacted positively with all the above pathogenic hubs, which would probably mean increase in the abundance of pathogens in the gut microbiome of T2DM patients. Between HC and DR, we had identified that 20 genera decreased in abundance but more interesting is the fact that 10 of 20 genera that decreased had anti-inflammatory characteristics (Table 4). Thus, one of the possible pathways influencing DR could be increased inflammation due to decrease in anti-inflammatory bacteria rather than an increase in pro-inflammatory genera. The interaction network in DR also indicated that some anti-inflammatory hub genera, *Butyrivibrio,* negatively correlated with another anti-inflammatory hub genus, *Megasphaera*. At the same time the hub genera, *Butyrivibrio*, interacted negatively with *Lactobacillus*, a probiotic hub genus, but positively with two pathogenic hub genera, *Cloacibacillus* and *Synergistes*. In contrast, the *Lactobacillus* and *Megasphaera* showed negative correlation with pathogenic hub genera, *Cloacibacillus* and *Synergistes*. Thus, it appears that in the DR interaction networks, the modulation appears to be working with decrease in anti-inflammatory genera, a decrease in probiotic bacteria and an increase in other bacteria.

In conclusion, (1) we confirm dysbiosis in the gut microbiomes of people with T2DM compared to healthy controls*;* (2) in first of such kind of study, we report that the gut microbiomes of people with DR differs from HC in the abundance of several different genera, which grouped separately in the heatmap and in the NMDS plots; (3) gut microbiomes of T2DM and DR did show significant differences at the genera level; (4) gut microbiome of DR patients was more discriminatory than T2DM patients compared to HC; (5) DR microbiomes showed a decrease in anti-inflammatory, probiotic and possibly pathogenic bacteria.

Novel therapeutics could emerge in future from the current information and future research on the gut microbiome in people with DM and DR. A specific pre- and/or pro-biotics could possibly delay the disease progression, if not reverse the disease process.

## Methods

### Ethics committee approval and subjects selected for this study

The study was approved by the Institutional Review Board and the Ethics Committee (Ref. No.LEC 12-15-122) of L. V. Prasad Eye Institute, Hyderabad, India; the study adhered to the tenets of Helsinki for research involving human subjects. The study recruited 3 groups of subjects: healthy human control (HC), people with T2DM without DR and people with T2DM and clinically manifest DR. All the T2DM and DR recruits were new recruits; in the HC cohort (n = 30) the gut microbiomes were generated from 17 new individuals and the remaining 13 HC individuals were taken from the controls of our earlier studies^16,19,20^. Individuals with no significant ocular and systemic pathology were recruited in the HC cohort: the T2DM cohort included subjects (a) positive for at least one of the three biochemical tests (HbA1c > 7%, fasting blood sugar > 120 mg% and post-prandial blood sugar > 200 mg%); (b) had history of taking anti-diabetic medications (Metformin or combinations of Metformin and / or Insulin) and (c) had no clinical signs of DR. The third cohort consisted of subjects confirmed to having DR based on the fundus examination / photograph followed by fundus fluorescein angiography (FFA) and optical coherence tomography (OCT). FFA and OCT were done only in people who had DR lesions in the fundus examination/photograph. Individuals who had undergone intraocular surgery or received intra-vitreal injections, implantable steroid within 90 days or having ocular or peri-ocular infection, uncontrolled glaucoma, presence of any form of ocular malignancy, had undergone gastrointestinal tract surgery or having kidney disease, cardiovascular disease, obesity, inflammatory bowel disease, prolonged constipation or diarrhoea and had taken any antibiotics, probiotics, or prebiotics 3 months prior to sample collection were excluded. Written informed consent was obtained from all study subjects prior to sample collection.

### Sample collection

Fecal samples (30 mg) were collected by the participants of all the 3 cohorts at home in a sterile container (HiMedia, India) and delivered within 4 h at LVPEI. Samples were frozen at − 80 °C for future extraction of DNA. The stool sample was homogenized with a sterile spatula and DNA extraction was done in duplicate using QIAamp DNA stool minikit (Qiagen, Hilden, Germany). Equal volume of DNA from each replicate was pooled and used for PCR amplification and sequencing. Quality of extracted DNA was checked on 0.8% agarose gel and quantified using Qubit 2.0 fluorometer with Qubit dsDNA HS Assay kit (Life Technologies, India).

### Amplification, illumina library preparation, and amplicon sequencing

V3–V4 region of bacterial 16S rRNA gene was amplified using 5′-CCTACGGGNGGCWGCAG-3′ and 5′-GACTACHVGGGTATCTAATCC-3′ primers. Subsequently, the bacterial amplicon libraries were prepared according to standard Illumina protocol and sequenced at Xcelris Genomics (Ahmedabad, India) using Illumina HiSeq 2 X 250 base pair chemistry.

### Taxonomy assignment of sequenced reads and removal of batch effect

Paired-end reads of each sample were assembled using FLASH software. Low quality sequences (average Phred score < 25) and chimeric sequences were removed with Prinseq-lite and Usearch61 respectively. Operational taxonomic unit (OTU) picking from the high quality reads was performed using `open reference OTU picking approach’ in QIIME (Quantitative Insights into Microbial Ecology) pipeline that used GreenGenes OTUs (V3V4) clustered at a 97% sequence similarity. Taxonomic assignments of the denovo-OTUs was accomplished using Wang Classifier^59^ with a bootstrap of 80%. OTUs representing < 0.001% of the total number of reads were assigned as sparse OTUs and were not included for further analysis.

Data was treated for batch effects using the ComBat function in package SVA^60^ to overcome variations between samples of the same cohort when they were analysed by NGS at different points of time using the same protocol and NGS platform. The DNA extraction and sequencing of the samples were done in two batches since the availability of the samples was dependent on the recruitment of subjects. Batch I included 13 HC (HC005–HC028), 11 T2DM (T2DM001–T2DM012) and 10 DR (DR002–DR013) samples and batch II included 17 HC (HC0037–HC053), 13 T2DM (T2DM013–T2DM025) and 18 DR (DR014–DR031) samples. Samples in Batch I and II were analysed together up to OTU picking and taxonomy assignment. Subsequently, the abundance table was split on the basis of cohorts and batch effect correction was applied to each cohort separately. Finally the batch effect corrected OTUs abundance was combined and used for all further analysis.

### Alpha diversity analyses of the microbiomes

Rarefaction curves and α (Alpha) diversity indices (Shannon diversity, Simpson index, number of observed OTUs, and Chao1 index) of the microbiomes were plotted using R-Vegan 2.4-2 package (http://vegan.r-forge.r-project.org/). Consequently, t-test was performed to analyse whether the α-diversity was significantly different between the 3 groups.

### Identification of differentially abundant taxonomic groups

Kruskal–Wallis and Wilcoxon tests were performed to identify the differentially abundant [Benjamini Hochberg (BH) corrected *p* < 0.05] taxonomic groups (at the phylum and genus level) in the bacterial microbiomes. Non-metric multidimensional scaling (NMDS) plots of microbiome samples were generated (using Bray–Curtis dissimilarity) based on OTUs and discriminatory genera.

### Inferring functional profiles of bacterial microbiomes

PICRUSt^61^ was used for inferring functional pathways of the bacterial microbiomes of HC, T2DM and DR. Reference OTUs were assigned with PICRUSt-compatible taxonomy using GreenGenes (v 13.5) database and then the KEGG pathways^62,63^ were predicted. Wilcoxon signed rank test was performed to identify the differentially abundant KEGG pathways between two groups (BH corrected *p* < 0.05).

### Interaction networks between bacterial genera in microbiomes

Separate interaction networks were generated based on pair-wise correlations between abundances of different bacterial genera in the 3 cohorts of microbiomes using CoNet in Cytoscape^64^. Spearman correlation coefficient (r) was used to obtain the pair-wise correlations between abundances of the bacterial genera.

## Supplementary Information

Supplementary Table 1.

Supplementary Table 2.

Supplementary Table 3.

Supplementary Table 4.

Supplementary Table 5.

Supplementary Information.

## Data Availability

All data generated or analysed during this study are included in this article (and its Supplementary Information files). Metagenomic sequencing reads can be accessed from National Center for Biotechnology Information (NCBI) BioProject accession ID PRJNA646010.

## References

[1] Federation, I. D. (International Diabetes Federation, Brussels, Belgium, 2019).

[2] Blaser MJ (2014). The microbiome revolution. J. Clin. Invest..

[3] Cho I, Blaser MJ (2012). The human microbiome: At the interface of health and disease. Nat. Rev. Genet..

[4] Kau AL, Ahern PP, Griffin NW, Goodman AL, Gordon JI (2011). Human nutrition, the gut microbiome and the immune system. Nature.

[5] Oresic M (2008). Dysregulation of lipid and amino acid metabolism precedes islet autoimmunity in children who later progress to type 1 diabetes. J. Exp. Med..

[6] Qin J (2012). A metagenome-wide association study of gut microbiota in type 2 diabetes. Nature.

[7] Sjostrom L (2004). Lifestyle, diabetes, and cardiovascular risk factors 10 years after bariatric surgery. N. Engl. J. Med..

[8] Tang WH, Kitai T, Hazen SL (2017). Gut microbiota in cardiovascular health and disease. Circ. Res..

[9] Tlaskalova-Hogenova H (2011). The role of gut microbiota (commensal bacteria) and the mucosal barrier in the pathogenesis of inflammatory and autoimmune diseases and cancer: Contribution of germ-free and gnotobiotic animal models of human diseases. Cell. Mol. Immunol..

[10] Shivaji S (2017). We are not alone: A case for the human microbiome in extra intestinal diseases. Gut Pathog..

[11] Shivaji S (2019). Connect between gut microbiome and diseases of the human eye. J. Biosci..

[12] Watane A, Cavuoto KM, Banerjee S, Galor A (2019). The microbiome and ocular surface disease. Curr. Ophthalmol. Rep..

[13] Horai R (2015). Microbiota-dependent activation of an autoreactive T cell receptor provokes autoimmunity in an immunologically privileged site. Immunity.

[14] Huang X (2018). Gut microbiota composition and fecal metabolic phenotype in patients with acute anterior uveitis. Invest. Ophthalmol. Vis. Sci..

[15] Jayasudha R (2019). Implicating dysbiosis of the gut fungal microbiome in uveitis, an inflammatory disease of the eye. Invest. Ophthalmol. Vis. Sci..

[16] Kalyana Chakravarthy S (2018). Dysbiosis in the gut bacterial microbiome of patients with uveitis, an inflammatory disease of the eye. Indian J. Microbiol..

[17] Shimizu J (2016). Bifidobacteria abundance-featured gut microbiota compositional change in patients with Behcet’s disease. PLoS ONE.

[18] de Paiva CS (2016). Altered mucosal microbiome diversity and disease severity in Sjögren syndrome. Sci. Rep..

[19] Jayasudha R (2018). Alterations in gut bacterial and fungal microbiomes are associated with bacterial Keratitis, an inflammatory disease of the human eye. J. Biosci..

[20] Kalyana Chakravarthy S (2018). Alterations in the gut bacterial microbiome in fungal Keratitis patients. PLoS ONE.

[21] Rowan S (2017). Involvement of a gut-retina axis in protection against dietary glycemia-induced age-related macular degeneration. Proc. Natl. Acad. Sci. U. S. A..

[22] Zinkernagel MS (2017). Association of the intestinal microbiome with the development of neovascular age-related macular degeneration. Sci. Rep..

[23] Kostic AD (2015). The dynamics of the human infant gut microbiome in development and in progression toward type 1 diabetes. Cell Host Microb..

[24] Tetz G, Brown SM, Hao Y, Tetz V (2019). Type 1 diabetes: An association between autoimmunity, the dynamics of gut amyloid-producing *E. coli* and their phages. Sci. Rep..

[25] Zhao G (2017). Intestinal virome changes precede autoimmunity in type I diabetes-susceptible children. Proc. Natl. Acad. Sci. U. S. A..

[26] Bhute SS (2017). Gut microbial diversity assessment of indian type-2-diabetics reveals alterations in eubacteria, archaea, and eukaryotes. Front. Microbiol..

[27] Larsen N (2010). Gut microbiota in human adults with type 2 diabetes differs from non-diabetic adults. PLoS ONE.

[28] Pandolfi C, Pellegrini L, Sbalzarini G, Mercantini F (1994). Obesity and insulin resistance. Minerva Med..

[29] Bellocchi C, Volkmann ER (2018). Update on the gastrointestinal microbiome in systemic sclerosis. Curr. Rheumatol. Rep..

[30] Everard A, Cani PD (2013). Diabetes, obesity and gut microbiota. Best Pract. Res. Clin. Gastroenterol..

[31] Giongo A (2011). Toward defining the autoimmune microbiome for type 1 diabetes. ISME J..

[32] Karlsson FH (2013). Gut metagenome in European women with normal, impaired and diabetic glucose control. Nature.

[33] Murri M (2013). Gut microbiota in children with type 1 diabetes differs from that in healthy children: A case-control study. BMC Med..

[34] Zhang X (2013). Human gut microbiota changes reveal the progression of glucose intolerance. PLoS ONE.

[35] Remely M (2014). Effects of short chain fatty acid producing bacteria on epigenetic regulation of FFAR3 in type 2 diabetes and obesity. Gene.

[36] Donath MY, Shoelson SE (2011). Type 2 diabetes as an inflammatory disease. Nat. Rev. Immunol..

[37] Jumas-Bilak E (2007). Acidaminococcus intestini sp. Nov., isolated from human clinical samples. Int. J. Syst. Evol. Microbiol..

[38] Leser T (2016). Probiotic strains of *Bifidobacterium adolescentis*. Conf. Proc. IPC.

[39] Martín R (2014). The commensal bacterium *Faecalibacterium prausnitzii* is protective in DNBS-induced chronic moderate and severe colitis models. Inflamm. Bowel Dis..

[40] Morgan XC (2012). Dysfunction of the intestinal microbiome in inflammatory bowel disease and treatment. Genome Biol..

[41] Załęski A, Banaszkiewicz A, Walkowiak J (2013). Butyric acid in irritable bowel syndrome. Prz Gastroenterol..

[42] Lukiw WJ (2016). Bacteroides fragilis lipopolysaccharide and inflammatory signaling in Alzheimer's disease. Front. Microbiol..

[43] Elbere I (2018). Association of metformin administration with gut microbiome dysbiosis in healthy volunteers. PLoS ONE.

[44] Sun L (2018). Gut microbiota and intestinal FXR mediate the clinical benefits of metformin. Nat. Med..

[45] de la Cuesta-Zuluaga J (2017). Metformin is associated with higher relative abundance of mucin-degrading Akkermansia muciniphila and several short-chain fatty acid-producing microbiota in the gut. Diabetes Care.

[46] Bryrup T (2019). Metformin-induced changes of the gut microbiota in healthy young men: Results of a non-blinded, one-armed intervention study. Diabetologia.

[47] Forslund K (2015). Disentangling type 2 diabetes and metformin treatment signatures in the human gut microbiota. Nature.

[48] Napolitano A (2014). Novel gut-based pharmacology of metformin in patients with type 2 diabetes mellitus. PLoS ONE.

[49] Wu H (2017). Metformin alters the gut microbiome of individuals with treatment-naive type 2 diabetes, contributing to the therapeutic effects of the drug. Nat. Med..

[50] Dao MC (2016). Akkermansia muciniphila and improved metabolic health during a dietary intervention in obesity: Relationship with gut microbiome richness and ecology. Gut.

[51] Chen SG (2016). Exposure to the functional bacterial amyloid protein curli enhances alpha-synuclein aggregation in aged Fischer 344 rats and *Caenorhabditis elegans*. Sci. Rep..

[52] Beli E (2018). Restructuring of the gut microbiome by intermittent fasting prevents retinopathy and prolongs survival in db/db mice. Diabetes.

[53] Hugenholtz F, de Vos WM (2018). Mouse models for human intestinal microbiota research: A critical evaluation. Cell. Mol. Life Sci..

[54] Sohail MU, Althani A, Anwar H, Rizzi R, Marei HE (2017). Role of the gastrointestinal tract microbiome in the pathophysiology of diabetes mellitus. J. Diabetes Res..

[55] Guilloteau P (2010). From the gut to the peripheral tissues: The multiple effects of butyrate. Nutr. Res. Rev..

[56] Zhou L (2018). Faecalibacterium prausnitzii produces butyrate to maintain Th17/Treg balance and to ameliorate colorectal colitis by inhibiting histone deacetylase 1. Inflamm. Bowel Dis..

[57] Van den Abbeele P (2013). Butyrate-producing Clostridium cluster XIVa species specifically colonize mucins in an in vitro gut model. ISME J..

[58] Bibbo S, Dore MP, Pes GM, Delitala G, Delitala AP (2017). Is there a role for gut microbiota in type 1 diabetes pathogenesis?. Ann. Med..

[59] Wang Q, Garrity GM, Tiedje JM, Cole JR (2007). Naïve Bayesian classifier for rapid assignment of rRNA sequences into the new bacterial taxonomy. Appl. Environ. Microbiol..

[60] Leek JT, Johnson WE, Parker HS, Jaffe AE, Storey JD (2012). The sva package for removing batch effects and other unwanted variation in high-throughput experiments. Bioinformatics (Oxford, England).

[61] Langille MGI (2013). Predictive functional profiling of microbial communities using 16S rRNA marker gene sequences. Nat. Biotechnol..

[62] Kanehisa M, Goto S (2000). KEGG: Kyoto encyclopedia of genes and genomes. Nucleic Acids Res..

[63] Kanehisa M, Sato Y, Kawashima M, Furumichi M, Tanabe M (2015). KEGG as a reference resource for gene and protein annotation. Nucleic Acids Res..

[64] Faust K, Raes J (2016). CoNet app: Inference of biological association networks using Cytoscape. F1000Res.

[65] Anand S, Kaur H, Mande SS (2016). Comparative in silico analysis of butyrate production pathways in gut commensals and pathogens. Front. Microbiol..

[66] Feng W, Ao H, Peng C (2018). Gut microbiota, short-chain fatty acids, and herbal medicines. Front. Pharmacol..

[67] Hiippala K, Kainulainen V, Kalliomäki M, Arkkila P, Satokari R (2016). Mucosal prevalence and interactions with the epithelium indicate commensalism of Sutterella spp. Front. Microbiol..

[68] Ko C-Y (2019). Gut microbiota in obstructive sleep apnea-hypopnea syndrome: Disease-related dysbiosis and metabolic comorbidities. Clin. Sci..

[69] Polansky O (2015). Important metabolic pathways and biological processes expressed by chicken cecal microbiota. Appl. Environ. Microbiol..

[70] Musher, D. M. in *Medical Microbiology, 4th edition* (ed S. Baron) Ch. 30 (University of Texas Medical Branch at Galveston, 1996). 21413252

[71] 72Du, X. *et al.* Microbial Community and Short-Chain Fatty Acid Mapping in the Intestinal Tract of Quail. *Animals (Basel)*. **10**, 1006, doi:10.3390/ani10061006 (2020). 10.3390/ani10061006 PMC7341218 32526858

[72] Hsiao A (2014). Members of the human gut microbiota involved in recovery from Vibrio cholerae infection. Nature.

[73] Opota O (2014). Bacteremia caused by Comamonas kerstersii in a patient with diverticulosis. J Clin Microbiol..

[74] 75Guentzel, M. N. in *Medical Microbiology, 4th edition* (ed S. Baron) Ch. 26, (University of Texas Medical Branch at Galveston, 1996). 21413252

[75] Jangi S (2016). Alterations of the human gut microbiome in multiple sclerosis. Nat Commun..

[76] 77Radolf, J. D. in *Medical Microbiology, 4th edition* (ed S. Baron) Ch. 36, (University of Texas Medical Branch at Galveston, 1996). 21413252

[77] Panthee S, Paudel A, Blom J, Hamamoto H, Sekimizu K (2019). Complete Genome Sequence of Weissella hellenica 0916–4-2 and Its Comparative Genomic Analysis. Front Microbiol..

[78] 79Yu, H. S. *et al.* Anti-Inflammatory Potential of Probiotic Strain Weissella cibaria JW15 Isolated from Kimchi through Regulation of NF-κB and MAPKs Pathways in LPS-Induced RAW 264.7 Cells. *J Microbiol Biotechnol*. **29**, 1022–1032, doi:10.4014/jmb.1903.03014 (2019). 10.4014/jmb.1903.03014 31216608

[79] Cancello R (2019). Effect of Short-Term Dietary Intervention and Probiotic Mix Supplementation on the Gut Microbiota of Elderly Obese Women. Nutrients..

[80] Liévin V (2000). Bifidobacterium strains from resident infant human gastrointestinal microflora exert antimicrobial activity. Gut.

[81] Pituch A, Walkowiak J, Banaszkiewicz A (2013). Butyric acid in functional constipation. Prz Gastroenterol..

[82] Patterson, M. J. in *Medical Microbiology, 4th edition* (ed S. Baron) Ch. 13, (University of Texas Medical Branch at Galveston, 1996). 21413252

[83] Chen C-C (2019). Antimicrobial activity of Lactobacillus species against carbapenem-resistant enterobacteriaceae. Front. Microbiol..

[84] Nagpal R (2018). Human-origin probiotic cocktail increases short-chain fatty acid production via modulation of mice and human gut microbiome. Sci. Rep..

[85] Num SM, Useh NM (2014). Clostridium: Pathogenic roles, industrial uses and medicinal prospects of natural products as ameliorative agents against pathogenic species. Jordan J. Biol.. Sci..

[86] Prod'homme M (2017). Cutaneous infection and bactaeremia caused by Erwinia billingiae: A case report. New Microbes New Infect..

[87] Goldstein EJC, Citron DM, Peraino VA, Cross SA (2003). Desulfovibrio desulfuricans bacteremia and review of human Desulfovibrio infections. J. Clin. Microbiol..

[88] Kloesel B, Beliveau M, Patel R, Trousdale RT, Sia IG (2013). Bulleidia extructa periprosthetic hip joint infection, United States. Emerg. Infect. Dis..

[89] Ramanan P, Barreto JN, Osmon DR, Tosh PK (2014). Rothia bacteremia: A 10-year experience at Mayo Clinic, Rochester, Minnesota. J. Clin. Microbiol..

[90] Domingo MC (2015). Cloacibacillus sp., a potential human pathogen associated with bacteremia in Quebec and New Brunswick. J. Clin. Microbiol..

[91] Moellering RC (1992). Emergence of Enterococcus as a significant pathogen. Clin. Infect. Dis..

[92] Kelly JP, Curhan GC, Cave DR, Anderson TE, Kaufman DW (2011). Factors related to colonization with Oxalobacter formigenes in U.S. adults. J. Endourol..

[93] Killackey SA, Sorbara MT, Girardin SE (2016). Cellular aspects of shigella pathogenesis: Focus on the manipulation of host cell processes. Front. Cell Infect. Microbiol..

