# Supplementary material for: Alterations in the gut bacterial microbiome in people with type 2 diabetes mellitus and diabetic retinopathy
Source: Sci Rep. 2021 Feb 2;11:2738. doi: 10.1038/s41598-021-82538-0 (PMC7854632; doi:10.1038/s41598-021-82538-0)
Supplement: Supplementary file 6 — Supplementary Information. [file 41598_2021_82538_MOESM6_ESM.doc]

Alterations in the Gut Bacterial Microbiome in People with Type 2 Diabetes Mellitus and Diabetic Retinopathy

Taraprasad Das^1^, Rajagopalaboopathi Jayasudha^2^, SamaKalyana Chakravarthy^2^, Gumpili Sai Prashanthi^2^, Archana Bhargava^3^, Mudit Tyagi^1^, Padmaja Kumari Rani^1^, Rajeev Reddy Pappuru^1^, Savitri Sharma^2^ and Sisinthy Shivaji^2*^

**Supplementary information content**

**Supplementary Figure S1.** Rarefaction analysis of gut microbiomes from healthy controls (HC, n =30), Type 2 Diabetes mellitus (T2DM, n= 24) and Diabetic Retinopathy (DR, n=28) individuals.

**Supplementary Figure S2.** Abundance of different bacterial phyla in the gut microbiomes of healthy controls (HC, n = 30), Type 2 Diabetes mellitus (T2DM, n = 24) and Diabetic Retinopathy (DR, n = 28) individuals. ‘‘Less abundant phyla’’ includes phyla with < 1% mean abundance.

**Supplementary Figure S3.** Abundance of bacterial genera in the gut microbiomes of healthy controls (HC, n = 30), Type 2 Diabetes mellitus (T2DM, n = 24) and Diabetic Retinopathy (DR, n = 28) individuals. ‘‘Less abundant genera’’ includes genera with < 1% mean abundance.

**Supplementary Figure S4.** Beta diversity analysis using NMDS plots based on Bray-Curtis dissimilarity of discriminating genera in the gut microbiomes of HC, T2DM and DR. The bacterial community appeared to vary significantly across HC, T2DM and DR (PERMANOVA, *p* = 0.001).

**Supplementary Table S1.** Relative abundances of bacterial OTUs from 82 fecal microbiomes of Healthy controls (HC, n = 30), Type 2 Diabetes mellitus (T2DM, n = 24) and Diabetic Retinopathy (DR, n = 28) patients. Sparse OTUs (with < 0.001% of total number of reads assigned to OTUs) were not included.

**Supplementary Table S2.** Median abundance (%) of bacterial genera in the gut microbiomes of Healthy controls (HC, n = 30), people with Type 2 Diabetes mellitus (T2DM, n = 24) and diabetic Retinopathy (DR, n = 28).

**Supplementary Table S3.** Log 2-fold change of the abundances of discriminatory KEGG pathways (BH corrected p < 0.05) observed between gut microbiomes of healthy controls (HC) and Type 2 Diabetes mellitus patients (T2DM).

**Supplementary Table S4.** Log 2-fold change of the abundances of discriminatory KEGG pathways (BH corrected p < 0.05) observed between gut microbiomes of healthy controls (HC) and Diabetic Retinopathy patients (DR).

**Supplementary Table S5.** Log 2-fold change of the abundances of discriminatory KEGG pathways (BH corrected p < 0.05) observed between gut microbiomes of Type 2 Diabetes mellitus (T2DM) and Diabetic Retinopathy patients (DR).

**Supplementary Table S6.** Alteration of bacterial genera / species associated with type 2 diabetes mellitus.

**
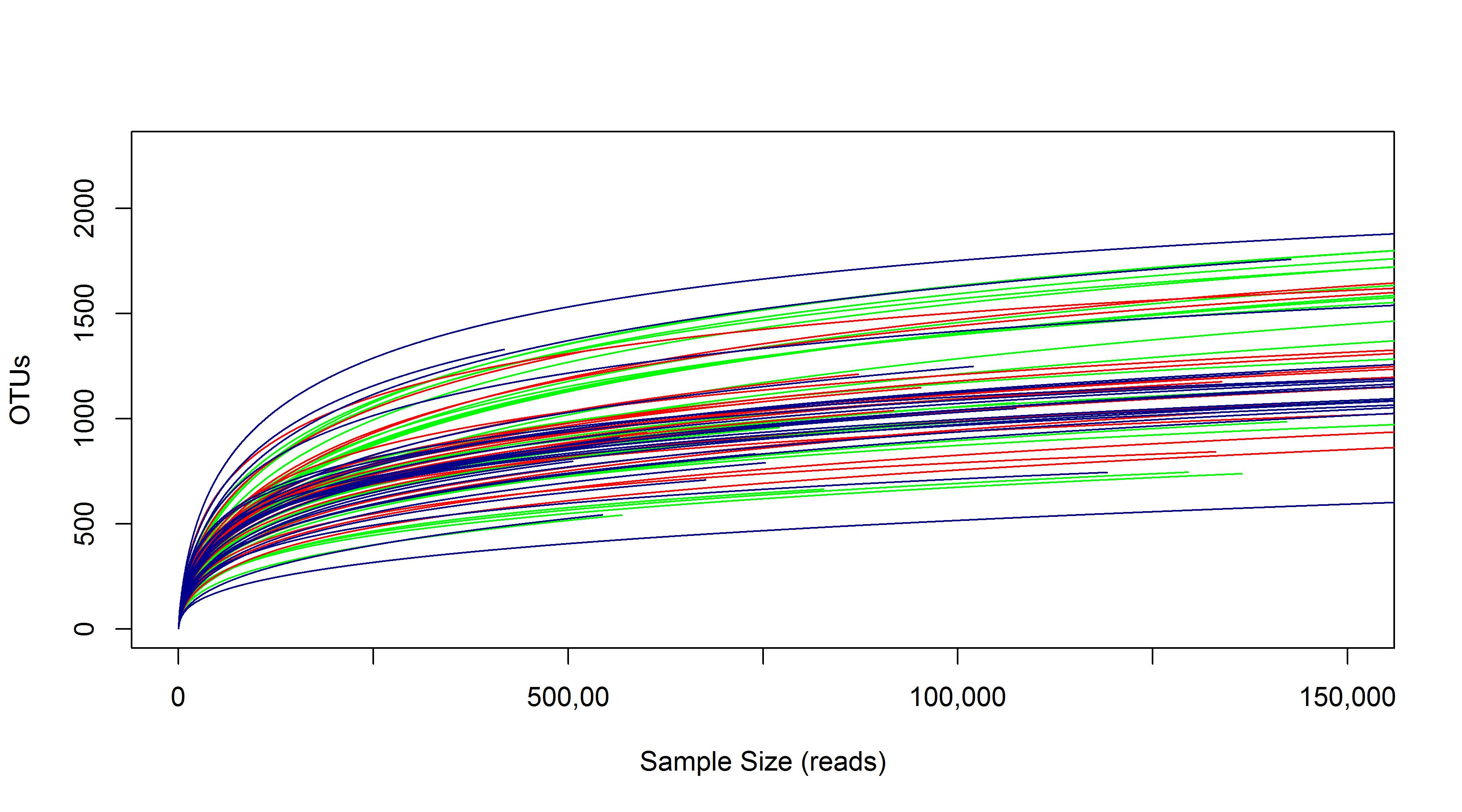
Supplementary Figure S1.** Rarefaction analysis of gut microbiomes from healthy controls (HC, n =30), Type 2 Diabetes mellitus (T2DM, n= 24) and Diabetic Retinopathy (DR, n=28) individuals.


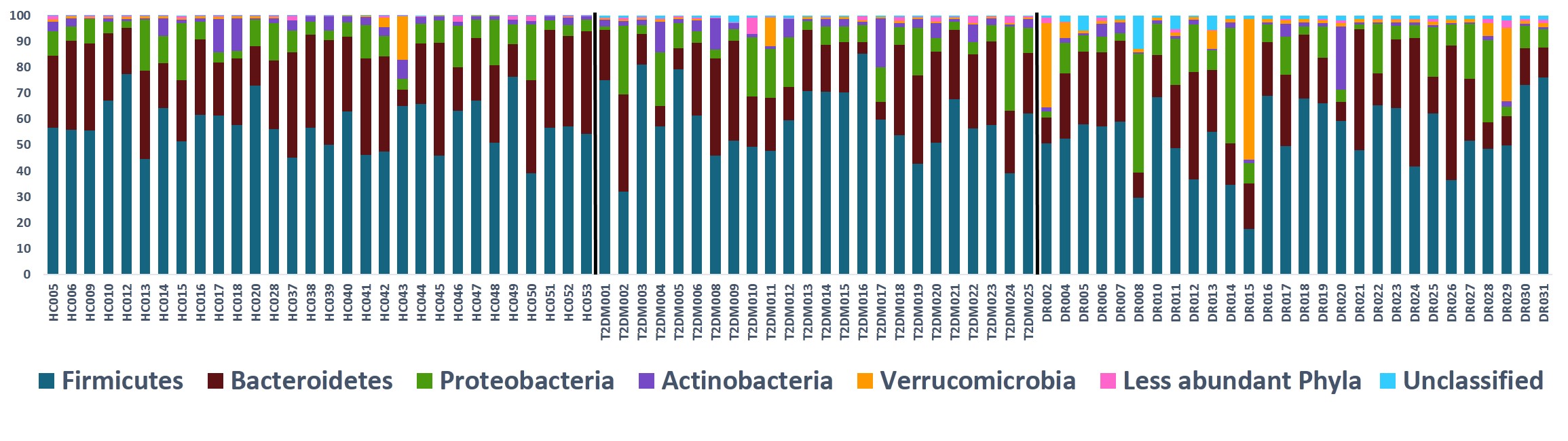


**Supplementary Figure S2.** Abundance of different bacterial phyla in the gut microbiomes of healthy controls (HC, n = 30), Type 2 Diabetes mellitus (T2DM, n = 24) and Diabetic Retinopathy (DR, n = 28) individuals. ‘‘Less abundant phyla’’ includes phyla with < 1% mean abundance.


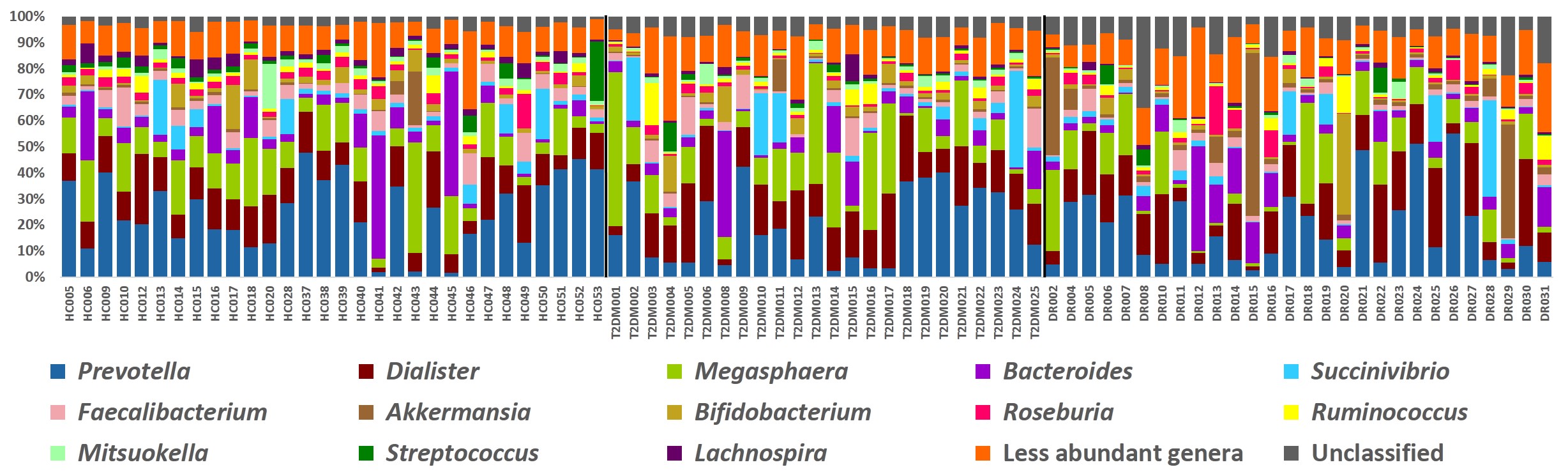


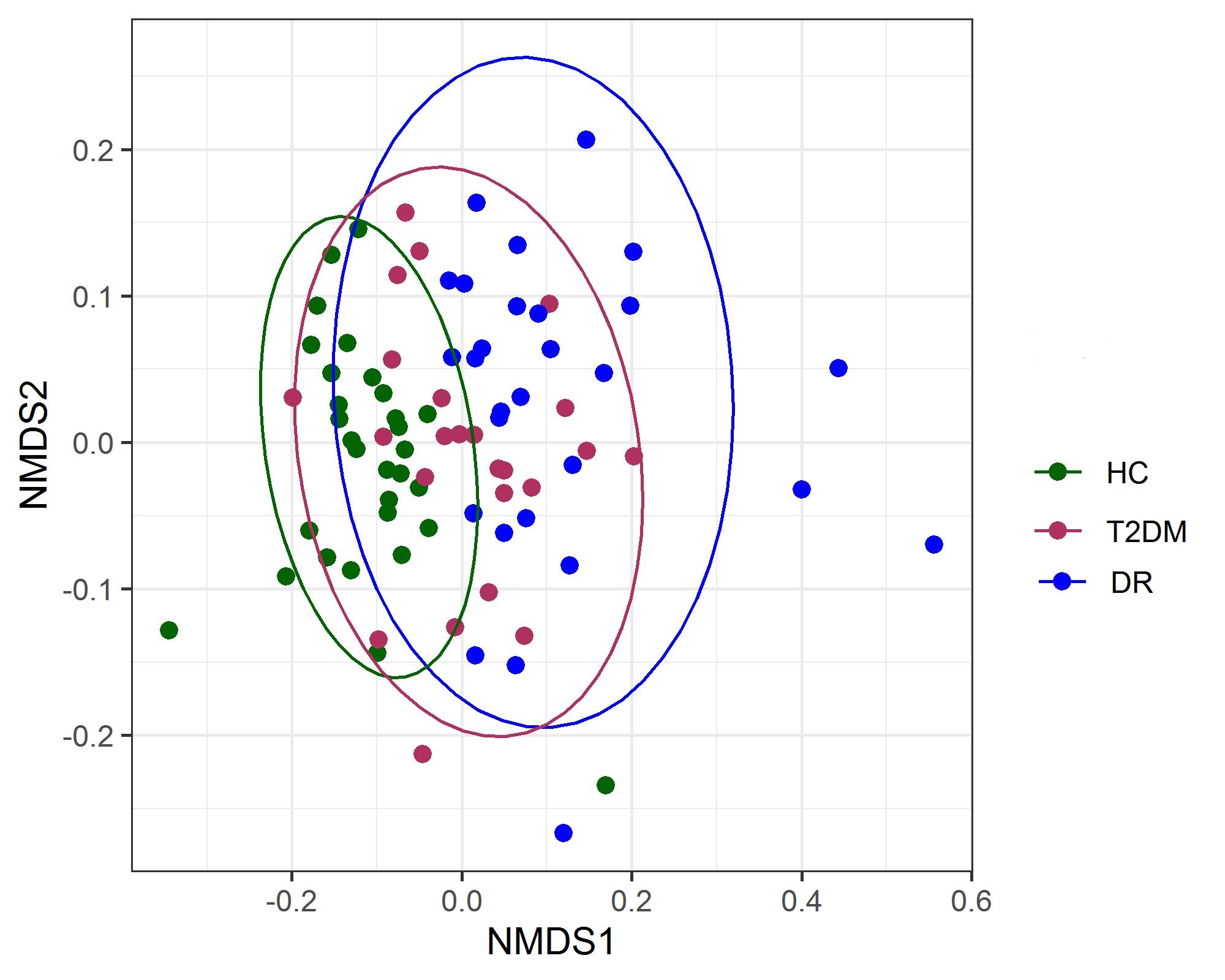
**Supplementary Figure S3.** Abundance of bacterial genera in the gut microbiomes of healthy controls (HC, n = 30), Type 2 Diabetes mellitus (T2DM, n = 24) and Diabetic Retinopathy (DR, n = 28) individuals. ‘‘Less abundant genera’’ includes genera with < 1% mean abundance.

**Supplementary Figure S4.** Beta diversity analysis using NMDS plots based on Bray-Curtis dissimilarity of discriminating genera in the gut microbiomes of HC, T2DM and DR. The bacterial community appeared to vary significantly across HC, T2DM and DR (PERMANOVA, *p* = 0.001).

**Supplementary Table S6.** Alteration of bacterial genera / species associated with type 2 diabetes mellitus

| **Microbiome** | **References** |
| --- | --- |
| ↑*Akkermansia muciniphila* | 6,30,34 |
| ↑*Bacteroides intestinalis*, ↑*Bacteroides sp.*  ↑*Bacteroides thetaiotaomicron*, ↓*Bacteroides vulgatus,*  ↑*Bacteroides ovatus*, ↓*Bacteroides fragilis* | 6,30,31,33,34 |
| ↓*Bifidobacterium* | 33 |
| ↓*Blautiacoccoides* | 33 |
| ↑*Clostridia*, ↓*Clostridium spp.* ↑*Clostridium bolteae*,  ↑*Clostridium ramosum*, ↑*Clostridium sp. HGF2*, ↑*Clostridium symbiosum*, ↑*Clostridium hathewayi*, ↑*Clostridium clostridioforme* | 6,31,32 |
| ↑*Desulfovibrio* sp. | 6 |
| ↑*Eggerthellalenta* | 6 |
| ↑*Escherichia coli* | 30 |
| ↓*Eubacterium*, ↓*Eubacterium rectale,*  ↑*Eubacterium* | 29,31,33 |
| ↓*Faecalibacterium,* ↓*Faecalibacterium prausnitzii,*  ↑*Faecalibacterium prausnitzii* | 31,34 |
| ↑*Lactobacillus* spp*.* | 31,32 |
| ↑*Prevotella* | 31 |
| ↓*Roseburia* | 32 |

↑increase; ↓decrease
